# Traditional Herbal Medicine: A Review of Potential of Inhibitory Hepatocellular Carcinoma in Basic Research and Clinical Trial

**DOI:** 10.1155/2013/268963

**Published:** 2013-07-11

**Authors:** Zhidong Wang, Jun Li, Yuanyuan Ji, Peng An, Shu Zhang, Zongfang Li

**Affiliations:** ^1^Department of General Surgery, The Second Affiliated Hospital, Xi'an Jiaotong University School of Medicine, Xi'an 710004, China; ^2^National Local Joint Engineering Research Center of Biodiagnostics and Biotherapy, Xi'an 710004, China; ^3^Shaanxi Province Engineering Research Center of Biotherapy and Translation Medicine, Xi'an 710004, China

## Abstract

Although significantly develops in hepatocellular carcinoma (HCC), features of HCC remain an aggressive cancer with a dismal outcome. Traditional Chinese medicine (TCM), specifically Chinese herbal medicine (CHM), is one of the most popular complementary and alternative medicine modalities worldwide. The use of heat-clearing and detoxicating (Chinese named *qingre jiedu*) CHM has attracted great attention as an alternative antitumor including HCC considering its low toxicity and high activity. Together these reports indicate that CHM is a promising anti-HCC herbal remedy in basic research. For patients with advanced HCC, CHM including formula and single combined with transcatheter arterial chemoembolization or chemotherapy is able to decrease tumor growth and the side effect of toxicity and improve overall survival, quality of life, and immune function. Due to its abundance, low cost, and safety in consumption, CHM remains a species with tremendous potential for further investigation in HCC.

## 1. Introduction

Hepatocellular carcinoma (HCC), one of the most common malignancies in China, ranks as the second leading cause of cancer mortality [1] and approximately accounts for half in the world [2]. Globally, there are approximately 750,000 new cases of HCC reported per year [3]. The incidence of HCC is increasing rapidly in the United States and other developed countries [3]. Moreover, features of HCC are an aggressive cancer with a dismal outcome largely due to metastasis and postsurgical recurrence [4].

As known, surgical resection, embolization, ablation, and chemotherapy play important roles in the treatment of HCC, but it is limited to a significant extent by its toxicities, significant resistance to available chemotherapeutic agents, side effects and complexities. Given the poor prognosis associated with HCC and limited treatment options outside of surgery, patients seek additional therapies to improve quality of life (QOL) or survival. One possible way to increase the efficacy of anticancer drugs and to decrease toxicities or side effects is to develop complementary and alternative medicine (CAM). Traditional Chinese medicine (TCM), one of the most popular CAM forms worldwide, has been widely used in cancer, especially from Chinese herbal medicine (CHM) [5]. 

In China, there has been a long history of using TCM in the treatment of liver cancer and other malignancies. The clinical diagnosis and treatment in TCM are mainly based on the *yin-yang* and five elements theories wood, fire, earth, metal, and water, an ancient philosophical concept used to explain the composition and phenomena of the physical universe. These theories apply the phenomena and laws of nature to the study of the physiological activities and pathological changes of the human body and its interrelationships. 

The typical TCM therapies include acupuncture, CHM, and *qigong* exercises. The CHM contains hundreds of medicinal substances, mainly plants, but also some minerals and animal products classified by their performed function in the body. Different parts of CHM such as the leaves, roots, stems, flowers, and seeds are widely used. Usually, CHM is combined in formulas and single. 

As the theory of TCM including CHM in the management of tumors concentrates on integrity and functional regulation of the organism, which coincides with oncology of modern medicine, its position in combination therapy for HCC has attracted more attention. Recently, accumulating evidences demonstrated that CHM attenuates HCC proliferation, invasion, and metastasis in basic research and improves patients with HCC survival and overall response rate as well in clinical study. This review will provide the reader with a new understanding of CHM's properties with an emphasis on regulation of multiple molecular targets of importance for HCC in basic research and representative CHM combined with classic treatment for HCC. 

## 2. The Representative CHM Formula in Basic Research

### 2.1. *Songyou Yin* Formula

Nontoxic and supporting the healthy energy (*Fuzheng Guben*) herbal formulas play active roles in the anticancer. *Songyou Yin*, one of the important representative *Fuzheng Guben *herbal formulas, consists of five herbs:* Radix Salviae Miltiorrhiae, Radix Astragali, Fructus Lycii*, *Fructus Crataegi,* and *Trionyx sinensis Wiegmann*. With respect to HCC invasion and metastasis,* Songyou Yin* inhibited the growth and invasion of HCC (MHCC97H) cells with high metastatic potential both *in vitro* and *in vivo* through inducing apoptosis and downregulation of MMP2 and VEGF expression [6]. Moreover, the formula improved living life, minimized cancer-related weight loss, and prolonged the survival of nude mice bearing tumors [6]. In experiments conducted for opposite effects of chemotherapy enhancing the malignancy of treated HCC, Xiong et al. found that *Songyou Yin *attenuated epithelial-mesenchymal transition (EMT) and inhibited the enhanced metastatic potential of residual HCC in nude mice [7]. For another, the formula can render HCC sensitive to oxaliplatin via the inhibition of stemness by induction of cancer stem cells (CSCs) differentiation or direct elimination of CSCs to decrease expression of CSC-related markers in tumor tissues and cell lines [8]. As known, palliative resection increased markedly HCC metastasis in patients with HCC. *Songyou Yin *reinforced the ability of IFN-*α* to inhibit palliative resection that induced the metastasis-enhancing potential by downregulation of VEGF and MMP2/TIMP2 [9].

### 2.2. *Jiedu Xiaozheng Yin* Formula

The use of heat clearing and detoxicating (Chinese named *qingre jiedu*) CHM including *Jiedu Xiaozheng Yin* has attracted great attention as an alternative antitumor including HCC. *Jiedu Xiaozheng Yin *(JXY), a polyherbal formula of TCM, consists of the following herbs *Hedyotis diffusa, Pseudobulbus Cremastrae seu Pleiones, Spica Prunellae *and *Radix Sophorae Flavescentis*. With regard to the study performed in HCC proliferation and growth, Cao et al. found that JXY inhibited the growth of HCC and the formation of cell colonies and blocked the cell cycle to G1 phase in a dose-dependent manner *in vitro*. Meanwhile, JXY showed an obvious antitumor effect *in vivo* through increasing the expression of G1-related proteins (cyclin D and cyclin E) [10]. In HCC angiogenesis, using human umbilical vein endothelial cells (HUVECs), chick chorioallantoic membrane (CCM), and an HCC mouse xenograft model, JXY treatment significantly reduced tube formation of HUVECs, and angiogenesis in the CCM, and microvessel density of tumor *in vivo* [11]. Further studies showed that JXY suppressed the vascular endothelial growth factor A (VEGF-A) expression and its receptor 2 (VEGFR-2) among the HCC HepG2 cells, HUVECs, and tumor [11]. About research played in hepatocarcinogenesis, Li et al. found that activation of c-fos proto-oncogene, c-jun, and c-myc oncogenes plays a crucial role in the pathogenesis of HCC [12]. 

### 2.3. *Huqi San* Formula

*Huqi San, *one of the experiences of famous Chinese anticancer drugs, consists of the following herbs *Ramulus Visci*, *Radix Astragali seu Hedysari*, *Radix Curcumaez* and *Radix Salviae Miltiorrhiae*. It demonstrated the inhibitory effect of it on rat befor-hepatocarcinogenesis induced by diethylnitrosamine (DEN) by analyzing the mutational activation of c-fos and overexpression of c-jun and c-myc. And the formula can inhibit the overexpression of it and liver preneoplastic lesions induced by DEN [12]. Another, Wen et al. demonstrated the modulation effect of energy metabolic enzyme expression on DEN-mediated hepatocarcinogenesis by* Huqi San*. They found that the formula treated rats showed significant decrease in areas of gamma-GT positive foci and activity of AFP [13]. On the other hand, the formula obviously increased these activities of G-6-Pase, SDH, and ATPase. *Huqi San* is a potential anticarcinogenic agent that may induce apoptosis by reducing the inhibitory effects of X-linked inhibitor of apoptosis protein on caspase-3 [14]. 

### 2.4. *Fuzheng Yiliu* Formula

*Fuzheng Yiliu* granule, one of the famous *Fuzheng Guben* herbal formulas in China, consists of four herbs: *Radix Hedysari, Radix Angelicae Sinensis, Radix Patriniae Scabrae*, and *Rhizoma Curcumae Phaeocaulis. *With respect to HCC immune function, the formula could inhibit HCC growth by regulating immune function and inducing apoptosis of tumor cells *in vivo* and *in vitro*. Cao et al. found that mice treated with *Fuzheng Yiliu* granule had higher percentages of CD3(+) and CD4(+), and more NK cells, and highest serum levels of IL-2 and TNF-*α* in the peripheral blood than those in the animals treated with normal saline. The results indicated that high-dose *Fuzheng Yiliu* granule-containing serum significantly decreased HepG2 cell viability, inhibited cell proliferation, and induced apoptosis [15]. The present study suggests that representative CHM formula properties with an emphasis on regulation of HCC multiple molecular targets are of importance *in vitro* and *in vivo*.

## 3. The Representative CHM Formula in Clinical Research

HCC is a multifactor and multistage process, and it is relatively difficultly to find a specific medication. Therefore, a wide consensus that combination therapy is most likely the way resulting in developing new therapy strategies for HCC in the future has been reached in academic institution. Transcatheter arterial chemoembolization (TACE) is the most widely used primary treatment for unresectable HCC because of its survival benefit, although its clinical effect is still far from satisfactory [16]. In China, there has been a long history of using CHM such as JDF granules, *Jiedu*, granules and *ganji *Decoction in the treatment of HCC and other malignancies. JDF granule preparation is composed of four CHM, root of *Actinidia valvata *and* Salvia chinensis*, bulb of *Cremastra appendiculata*, and *gizzard* membrane of *Gallus gallus domesticu* [17]. To evaluate the effect of combined therapy with TACE and JDF granules preparation in the treatment of patients with unresectable HCC on survival, current study data demonstrated that TACE combined with JDF granule preparation could improve the prognosis of patients and prolong the survival of patients with unresectable HCC [17]. Furthermore, the trial found that the median overall survival was 9.2 months (95% confidence interval [95% CI], 6.94–1.46) in the TACE combination with JDF formula versus 5.87 months (95% CI, 4.21–7.52) in the control group. In the TACE combination with JDF formula, 1-, 2-, and 3-year survival rates were 41.2%, 18.4%, and 9.6%, respectively. Additional, the Cox regression analysis revealed the therapy model to be an independent predictor of patient prognosis. Importantly, the current study provides preliminary and powerful data to support future evaluation of CHM in combination therapy for HCC in a large-cohort, randomized clinical trial (RCT). 

To effetely prevent the recurrence of HCC after surgical resection, a case-control trial, *Jiedu* granules, a CHM compound, plus cinobufacini injection, extracted from skin of *Bufo bufo gargarizans Cantor* after operation versus TACE after operation, was performed. *Jiedu* Granules is composed of CHM,* Herba Salviae Chinensis, Radix Actinidiae valvatae, Semen Coicis, Fructus Crataegi, Massa Medicata Fermentata, *and *Fructus Hordei Germinatus. *The trials demonstrated that *Jiedu* granules plus cinobufacini injection, a combination that is commonly used for postoperation management of HCC, can postpone tumor recurrence and metastasis, prolong the survival time, and increase the survival rate of postsurgical patients with HCC [18].

To evaluate whether CHM improves immune response for 1,008 unresectable HCC after TACE by using meta-analysis of data from the literature involving available 12 RCT of CHM in combination with TACE compared with that of TACE alone, literature retrieval was conducted. Meng et al. showed that the differences of pooled weighted mean difference before and after treatment and 95% CI were 13.63 (8.96–18.69; *P* = 0.0001) for the proportion of CD3(+) T cells, 10.56 (6.91–14.21; *P* = 0.0001) for the proportion of CD4(+) T cells, 3.40 (6.83 to 0.03; *P* = 0.052) for the proportion of CD8(+) T cells, 0.54 (0.42–0.66; *P* = 0.0001) for the ratio of CD4^+^/CD8^+^, and 12.34 (7.26–17.41; *P* = 0.0001) for the proportion of NK cells. No serious adverse events were reported. Thus, TCM in combination with TACE improves the immune response of patients with unresectable HCC [19]. To observe the efficacy of TCM comprehensive therapeutic project in treating the middle/late stage patients with HCC, with prospective RCT, 97 patients with HCC were assigned to the test group (49 cases) treated with TCM comprehensive therapy using *Oleum fructus bruceas *intervention combining oral intake of *ganji *Decoction and external application of *Ailitong,* and the control group (48 cases) was treated with chemotherapeutic agents combining iodized oil chemo-embolization and analgesics [20]. To compare the efficacy and safety of CHM plus TACE with that of TACE alone (therapy I versus therapy II) in treating unresectable HCC via a meta-analysis of all available 37 RCTs involving 2653 patients, an important research was performed [21]. The results showed that therapy I compared with therapy II improved the patient survival, QOF, alleviation of symptoms, and tumor response and was thus more therapeutically beneficial. Moreover, no serious adverse events were reported [21]. This trial study displayed that TCM comprehensive therapy is an effective treatment for the middle/late stage patients with HCC, and it could extend the pain-relieving sustained time and improve the patients' QOL and long-term survival with less adverse reaction. Evidence from patients with HCC trials suggests that representative CHM formula combined with TACE improve overall survival, QOL, and immune function. 

## 4. The Representative CHM Single Herb in Basic Research

CHM, including animal and plant herbs, plays important roles in the treatment of malignancy including HCC. Based on TCM theory, heat-clearing and detoxicating (Chinese named *qingre jiedu*) CHM such as toad's skin (*Chan chu pi*), *Flos Chrysanthemi Indici*, *Herba Scutellariae Darbatae Radix, Radix Sophorae Flavescentis,* and *Radix Scutellariae *has increasingly become important for treatment of HCC. Bufalin, one of the representative animal herbs, is the major component of *Chan-Chu* extracted from toad's skins. With respect to autophagy in HCC, Tsai et al. investigated the pharmacological mechanisms of cell cycle arrest and autophagic cell death induced by bufalin in SK-HEP-1 human HCC cells *in vitro* [22]. Data showed that bufalin triggered autophagic cell death and G2/M phase arrest through the AKT/mTOR signaling pathway in SK-HEP-1 cells. For another, bufalin triggered autophagy and enhanced Beclin-1 expression, LC3-I to LC3-II conversion, as well as decreased p62 expression and mTOR signaling activation in HCC HepG2 cells [23]. Blockage of autophagy by selective inhibitor 3-MA decreased apoptotic ratio in bufalin-treated HepG2 cells, suggesting that bufalin induces HepG2 cells for autophagy at least in part via AMPK-mTOR dependent pathway. Similarly, arenobufagin, a bufadienolide from toad venom, had potent antineoplastic activity against HCC HepG2 cells as well as corresponding multidrug-resistant HepG2/ADM cells. Arenobufagin induced mitochondria-mediated apoptosis in HCC cells as well as increasing Bax/Bcl-2 expression ratio. They observed the inhibition of phosphatidylinositol 3-kinase (PI3K)/Akt/mammalian target of rapamycin (mTOR) pathway by arenobufagin. Interestingly, inhibition of mTOR by rapamycin or siRNA duplexes augmented the arenobufagin-induced apoptosis and autophagy. Based on the evidenced above, underlying antineoplastic mechanisms of arenobufagin involve cross talk between apoptosis and autophagy via inhibition of the PI3K/Akt/mTOR pathway [24]. Regarding HCC angiogenesis, arenobufagin inhibited VEGF-induced viability, migration, invasion, and tube formation in HUVECs *in vitro* and suppressed sprouting formation from VEGF-treated aortic rings in an *ex vivo* model [25]. Furthermore, arenobufagin blocked angiogenesis in a matrigel plugs assay in HCC cells. Additionally, arenobufagin inhibited VEGF-induced VEGFR-2 autophosphorylation and suppressed the activity of VEGFR-2-mediated signaling cascades. With regard to HCC cell apoptosis and proliferation, our teams previous study showed that* Flos Chrysanthemi Indici *exerted a significant apoptotic effect via a mitochondrial pathway and arrested the cell cycle by regulation of cell cycle-related proteins in human MHCC97H cells, a HCC cell line with high metastatic potential [26]. Following, we investigated the effect of this herbon isoproterenol induced growth of human HCC cells in correlation with the intracellular activity of MAPK/ERK1/2 and found that the herb was effective in attenuating the mitogenic effect of isoproterenol on both HepG2 and MHCC97H cells. The inhibitory effect of the plant was mediated by inhibiting the isoproterenol-induced activation of MAPK/ERK1/2 via beta2-AR in tumor cells [27]. In addition, our study indicated that *Flos Chrysanthemi Indici *inhibited the invasion and migration ability of MHCC97H cells, as assesses through a matrigel-coated membrane invasion. Moreover, the herb could reduce the cancer cell metastatic ability, in part at least, through decrease of the MMP expression, simultaneous increase of the TIMP expression, and further restoring their balance as a therapeutic target in HCC [28]. Dai et al. found that *Herba Scutellariae Darbatae Radix *(HSDR) effectively inhibits the proliferation and induces apoptosis of H22 cells involving loss of mitochondrial transmembrane potential, release of cytochrome C, and activation of caspase-3 in a dose-dependent manner [29].The effect of HSDR on immune function was determined using hepatoma H22 tumor bearing mice. The results demonstrate that the plant increased weight, thymus, and spleen index of H22 bearing mice and phagocytotic function of macrophages by observing peritoneal macrophages phagocytize chicken red blood cells. It is suggested that HSDR improves the immune function of hepatoma H22 tumor bearing mice [30]. Regarding the detoxification and enhancement for chemotherapy, high-dose HSDR combined with 5-fluorouracil (5-FU) could significantly enhance the tumor inhibition rate, thymus, and spleen index in immunological organs. The data displayed that HSDR can significantly enhance the tumor inhibition rate of 5-FU, reduce the toxic effects, prolong the survival time, and improve immune function in the H22 tumor-bearing mice [31]. Matrine, an alkaloid purified from the Chinese herb *Radix Sophorae Flavescentis,* significantly inhibited the proliferation and induced G1-phase cell cycle arrest and apoptosis of HCC HepG2 cells in a dose-dependent manner. Fewer autophagic vacuoles were observed in the combined 3-MA and matrine treatment group when 3-MA was added before matrine treatment, indicating that both autophagy and apoptosis are activated when matrine-induced death of HepG2 cells occurs [32]. Baicalein, a purified flavonoid extracted from the roots of *Radix Scutellariae*, exhibited significant cytotoxicity to three HCC cell lines but marginal cytotoxicity to a normal liver cell line. Treatment with baicalein dramatically reduced mitochondrial transmembrane potential, activated caspase-9 and caspase-3, and significantly inhibited tumor growth of HCC xenografts in mice. Furthermore, Baicalein treatment dramatically decreased the levels of phosphorylation of MEK1, ERK1/2, and Bad *in vitro* and *in vivo*. Consequently, these findings suggest that Baicalein preferentially inhibits HCC tumor growth through inhibition of MEK-ERK signaling and by inducing intrinsic apoptosis [33]. These results suggest the representative CHM single herb properties with an emphasis on regulation of HCC multiple molecular targets of importance *in vitro* and *in vivo*.

## 5. The Representative CHM Single Herb in Clinical Research

*Kanglaite* injection, one of Chinese herb preparations, extracted from CHM coix seed oil, had been confirmed with antitumor activity including HCC [34]. Zhu et al. found that *Kanglaite *injection combined with TACE was significantly superior to TACE group in improving symptoms and Karnofsky scores, decreasing tumor growth and side-effect of marrow toxicity for patients with advanced stage HCC [35]. Regarding the single CHM use in combination with TACE, occasionally, single CHM was used to be combined with some first-line anticancer agents. *Kanglaite *injection reverses the multidrug resistance and has obvious chemotherapy sensitization [36]. Thus, to observe the therapeutic effect and safety, several researchers focused on studying the combination form of CHM and some chemotherapeutic agents. For instance, compared with Capecitabine group, *Kanglaite* injection combined with Capecitabine for patients with advanced stage HCC displayed better clinical benefit response and tolerance, longer time-to-progression, and median survival time [36]. *Fructus Bruceae* oil and *ganji* recipe combination of TACE showed that the improvements in the treatment group, including patients' physical energy enhancing, symptoms alleviating, and overall QOL improving, were superior to those in the TACE group [37]. Yang et al. observed that patients with advanced HCC were treated with TACE using B. javanica oil/lipiodol mixture perfusion remobilization (treatment group) or using conventional chemotherapy/lipiodol chemoembolization infusion (control group) [38]. The data displayed that the incidence of toxic side effects in the treatment group was less than that in the control group after the intervention. Compare with the 0.5 and 1-year survival rate, the treatment group was 96. 1% (25/26) and 50% (13/26) and the control group was 83.3% (20/24) and 33.3% (8/24). Additionally, two group cases with a median survival time were 8.9 and 6.7 months, respectively. It is suggests that representative single CHM combined with TACE improved the overall survivals, QOL and reduces the toxic side effects. 

## 6. The Effects of Detoxification and Enhancement for Chemotherapy on CHM

Previous studies have demonstrated that, one of the representative CHM, HSDR increased the weight, thymus, and spleen index of H22 bearing mice and phagocytotic function of macrophages by observing peritoneal macrophages phagocytize chicken red blood cells [31]. Regarding the detoxification and enhancement for chemotherapy, high-dose HSDR combined with 5-FU could significantly enhance the tumor inhibition rate, thymus, and spleen index in immunological organs. The data displayed that CHM can significantly reduce the toxic effects and improve immune function. CHM plus TACE significantly increases nondeterioration performance, T cells, natural killer cells, and white blood cell count, significantly decreases the level of blood alpha-fetoprotein concentration, and significantly lowers the risk for nausea and vomiting [39]. Furthermore, meta-analysis has also demonstrated that CHM plus TACE increases the proportions of cluster differentiation CD3(+) T cells, CD4(+) T cells, and NK cells, as well as the ratio of CD4^+^/CD8^+^ before and after treatment [19]. A meta-analysis of RCT for CHM combined with chemotherapy has reported promising evidence that CHM plus chemotherapy may have benefits on patients with HCC [40]. *Astragalus *might also boost host immune function by stimulating macrophage and NK cell activity [31]. Moreover, *Astragalus *appears to restore *in vitro *T-cell function, which is suppressed in cancer patients [31]. The main ingredient of *Kanglaite* injection is coix seed oil.* Kanglaite* injection exerted a significant inducing apoptotic effect and arrested the cell cycle by inhibition of M stage in cancer cells. Another, *Kanglaite* injection actives NK cells, promotes phagocytosis, and improves immune function as well [34, 35]. And it can induce tumor cell apoptosis, block tumor cell mitosis, improve immune function, reduce the toxicity of chemotherapy, and relieve cancer pain [34]. *Fructus Bruceae* oil emulsion (*Yadanzi *oil), one of Chinese herb preparations, can significantly inhibit topoisomerase, reverses the multidrug resistance, and has obvious chemotherapy sensitization in HCC cells [37].

## 7. Side Effects of CHM

No significant drug-related side effects were observed in the study group. The majority of patients reported mild to moderate side effects. The main side effects associated with CHM were lower fever, gastrointestinal symptoms, bone marrow suppression, neurotoxicity, hand-foot syndrome, and so forth [17, 34–38]. 

## 8. Conclusions

The rising of the use of TCM, particularly CHM has worldwide sparked an extreme interest in more and more people and researchers. Recently, the use of heat-clearing and detoxicating (Chinese named *qingre jiedu*) CHM has attracted great attention as an alternative antitumor including HCC considering its low toxicity and high activity. For HCC in basic research, CHM has been found to be active against hepatocarcinogenesis, proliferation, invasion, metastasis, and angiogenesis. Additionally, CHM combined with chemotherapy could significantly enhance the HCC inhibition rate and immune function in immunological organs and reduce the toxic effects. All these beneficial effects will ameliorate the radiation, chemotherapy, and TACE induced adverse effects. These outcomes of such studies may be useful for the clinical applications of HCC in human beings against cancers and may open up a new therapeutic avenue. Due to its abundance, low cost, and safety in consumption, CHM remains a species with tremendous potential for further investigation in HCC. 

For patients with advanced HCC, CHM including formula and single was used to be combined with TACE or chemotherapy usually, decreasing tumor growth and side effect of toxicity, and to prolong overall survival, QOF, and immune function. Together these reports indicate that CHM is a promising anti-HCC herbal remedy. However, due to various factors—including inconsistencies in treatment schemes, the limited sampling sizes, and lack of quality assurance of the herbal products well-designed RCTs to prove the effectiveness of TCM as adjuvant therapy for cancer are scarce. In general, most of the published clinical studies were trials without rigorous randomization or they involved single group pre-post, cohort, time series, or matched case-control studies. Fore another, studies should be performed on a large number of HCC patients receiving radiotherapy, chemotherapeutic, postoperative combination with CHM. Moreover, the clinical trials are insufficient to draw firm conclusions for the single use of CHM in patients with HCC.

## References

[1] Huang GW, Tao YM, Ding X (2009). Endocan expression correlated with poor survival in human hepatocellular carcinoma. *Digestive Diseases and Sciences*.

[2] Ni Y, Gong XG, Lu M, Chen HM, Wang Y (2008). Mitochondrial ROS burst as an early sign in sarsasapogenin-induced apoptosis in HepG2 cells. *Cell Biology International*.

[3] Maluccio M, Covey A (2012). Recent progress in understanding, diagnosing, and treating hepatocellular carcinoma. *CA: A Cancer Journal For Clinicians*.

[4] Zhu K, Dai Z, Pan Q (2011). Metadherin promotes hepatocellular carcinoma metastasis through induction of epithelial-mesenchymal transition. *Clinical Cancer Research*.

[5] Yang G, Li X, Li X (2012). Traditional Chinese medicine in cancer care: a review of case series published in the Chinese literature. *Evidence Based Complementary and Alternative Medicine*.

[6] Huang XY, Wang L, Huang ZL, Zheng Q, Li QS, Tang ZY (2009). Herbal extract Songyou Yin inhibits tumor growth and prolongs survival in nude mice bearing human hepatocellular carcinoma xenograft with high metastatic potential. *Journal of Cancer Research and Clinical Oncology*.

[7] Xiong W, Ren ZG, Qiu SJ (2010). Residual hepatocellular carcinoma after oxaliplatin treatment has increased metastatic potential in a nude mouse model and is attenuated by Songyou Yin. *BMC Cancer*.

[8] Jia QA, Ren ZG, Bu Y (2012). Herbal compound, “Songyou Yin” renders hepatocellular carcinoma sensitive to oxaliplatin through inhibition of stemness. *Evidence-Based Complementary and Alternative Medicine*.

[9] Huang XY, Huang ZL, Wang L (2010). Herbal compound “Songyou Yin” reinforced the ability of interferon-alfa to inhibit the enhanced metastatic potential induced by palliative resection of hepatocellular carcinoma in nude mice. *BMC Cancer*.

[10] Cao Z, Lin W, Huang Z (2013). Ethyl acetate extraction from a Chinese herbal formula, Jiedu Xiaozheng Yin, inhibits the proliferation of hepatocellular carcinoma cells via induction of G0/G1 phase arrest in vivo and in vitro. *International Journal of Oncology*.

[11] Cao Z, Lin W, Huang Z (2013). Jiedu Xiaozheng Yin, a Chinese herbal formula, inhibits tumor angiogenesis via downregulation of VEGF-A and VEGFR-2 expression in vivo and in vitro. *Oncology Reports*.

[12] Li X, Shi ZM, Feng P, Wen ZY, Wang XJ (2007). Effect of Qi-protecting powder (Huqi San) on expression of c-jun, c-fos and c-myc in diethylnitrosamine-mediated hepatocarcinogenesis. *World Journal of Gastroenterology*.

[13] Wen Z, Shi Z, Feng P, Xue X, Dong K, Wang X (2008). Modulation of energy metabolic enzyme expression in N-nitrosodiethylamine-mediated hepatocarcinogenesis by Chinese herbs, Huqi San. *BioFactors*.

[14] Zeng X, Li X, Xue X (2013). Activation of apoptosis in hepatocellular carcinoma by the Chinese traditional medicine Hu Qisan. *Experimental and Therapeutic Medicine*.

[15] Cao ZY, Chen XZ, Liao LM (2011). Fuzheng Yiliu Granule inhibits the growth of hepatocellular cancer by regulating immune function and inducing apoptosis in vivo and in vitro.. *Chinese Journal of Integrative Medicine*.

[16] Takayasu K (2013). Transcatheter arterial chemoembolization for unresectable hepatocellular carcinoma: recent progression and perspective. *Oncology*.

[17] Yu Y, Lang Q, Chen Z (2009). The efficacy for unresectable hepatocellular carcinoma may be improved by transcatheter arterial chemoembolization in combination with a traditional Chinese herbal medicine formula: a retrospective study. *Cancer*.

[18] Chen Z, Chen HY, Lang QB (2012). Preventive effects of jiedu granules combined with cinobufacini injection versus transcatheter arterial chemoembolization in post-surgical patients with hepatocellular carcinoma: a case-control trial. *Chinese Journal of Integrative Medicine*.

[19] Meng MB, Wen QL, Cui YL, She B, Zhang RM (2011). Meta-analysis: traditional Chinese medicine for improving immune response in patients with unresectable hepatocellular carcinoma after transcatheter arterial chemoembolization. *Explore*.

[20] Tian HQ, Li HL, Wang B (2010). Treatment of middle/late stage primary hepatic carcinoma by Chinese medicine comprehensive therapy: a prospective randomized controlled study. *Chinese Journal of Integrative Medicine*.

[21] Meng MB, Cui YL, Guan YS (2008). Traditional Chinese medicine plus transcatheter arterial chemoembolization for unresectable hepatocellular carcinoma. *Journal of Alternative and Complementary Medicine*.

[22] Tsai SC, Yang JS, Peng SF (2012). Bufalin increases sensitivity to AKT/mTOR-induced autophagic cell death in SK-HEP-1 human hepatocellular carcinoma cells. *International Journal of Oncology*.

[23] Miao Q, Bi LL, Li X, al et (2013). Anticancer effects of bufalin on human hepatocellular carcinoma HepG2 cells: roles of apoptosis and autophagy. *International Journal of Molecular Sciences*.

[24] Zhang DM, Liu JS, Deng LJ (2013). Arenobufagin, a natural bufadienolide from toad venom, induces apoptosis and autophagy in human hepatocellular carcinoma cells through inhibition of PI3K/Akt/mTOR pathway. *Carcinogenesis*.

[25] Li M, Wu S, Liu Z (2012). Arenobufagin, a bufadienolide compound from toad venom, inhibits VEGF-mediated angiogenesis through suppression of VEGFR-2 signaling pathway. *Biochemical Pharmacology*.

[26] Li ZF, Wang ZD, Ji YY (2009). Induction of apoptosis and cell cycle arrest in human HCC MHCC97H cells with *Chrysanthemum indicum* extract. *World Journal of Gastroenterology*.

[27] Yuan A, Li Z, Li X (2009). Distinct effect of *Chrysanthemum indicum* Linné extracts on isoproterenol-induced growth of human hepatocellular carcinoma cells. *Oncology Reports*.

[28] Wang ZD, Huang C, Li ZF (2010). *Chrysanthemum indicum* ethanolic extract inhibits invasion of hepatocellular carcinoma via regulation of MMP/TIMP balance as therapeutic target. *Oncology Reports*.

[29] Dai ZJ, Wang XJ, Li ZF (2008). *Scutellaria barbate* extract induces apoptosis of hepatoma H22 cells via the mitochondrial pathway involving caspase-3. *World Journal of Gastroenterology*.

[30] Dai ZJ, Gao J, Li ZF (2011). In vitro and in vivo antitumor activity of *Scutellaria barbate* extract on murine liver cancer. *Molecules*.

[31] Dai Z, Liu X, Ji Z (2008). The effect-enhancing and toxicity-reducing action of the extract of Herba *Scutellariae barbatae* for chemotherapy in hepatoma H22 tumor-bearing mice. *Journal of Traditional Chinese Medicine*.

[32] Zhang JQ, Li YM, Liu T (2010). Antitumor effect of matrine in human hepatoma G2 cells by inducing apoptosis and autophagy. *World Journal of Gastroenterology*.

[33] Liang RR, Zhang S, Qi JA (2012). Preferential inhibition of hepatocellular carcinoma by the flavonoid Baicalein through blocking MEK-ERK signaling. *International Journal of Oncology*.

[34] Zhan YP, Huang XE, Cao J (2012). Clinical safety and efficacy of Kanglaite (Coix Seed Oil) injection combined with chemotherapy in treating patients with gastric cancer. *Asian Pacific Jounal of Cancer Prevention*.

[35] Zhu XF (2006). The clinical observation of the effect of kanlaite injection combined with chemoembolization primary on middle and advanced stage liver cancer. *Jounal of Basic and Clinical Oncology*.

[36] Xu XH, Su J, Fu XY (2010). Kanlaite effect of injection combination with capecitabin on patients with advanced stage HCC. *Li Shizhen Medicine and Materia Medica Research*.

[37] Wang B, Tian HQ, Liang GW (2009). Effect of ganji recipe combined with Fructus Bruceae oil emulsion intervention on quality of life in patients with advanced primary hepatic cancer. *Chinese Journal of Integrated Traditional and Western Medicine*.

[38] Yang MZ, Wang H, Jiang GJ, Zhao KF, Cai Z, Zhao K (2011). Interventional treatment for elderly advanced primary liver cancer by *Brucea javanica* Oil emulsion. *Chinese Journal of Experimental Traditional Medical Formulae*.

[39] Liao YH, Lin CC, Li TC, Lin JG (2012). Utilization pattern of traditional Chinese medicine for liver cancer patients in Taiwan. *BMC Complementary and Alternative Medicine*.

[40] Shu X, McCulloch M, Xiao H, Broffman M, Gao J (2005). Chinese herbal medicine and chemotherapy in the treatment of hepatocellular carcinoma: a meta-analysis of randomized controlled trials. *Integrative Cancer Therapies*.

